# Aluminium oxide nanoparticles compromise spatial memory performance and proBDNF-mediated neuronal function in the hippocampus of rats

**DOI:** 10.1186/s12989-022-00477-8

**Published:** 2022-05-10

**Authors:** Wei Sun, Jia Li, Xiaoliang Li, Xiao Chen, Yazi Mei, Yang Yang, Lei An

**Affiliations:** 1 Department of Pediatric, The First Affiliated Hospital, Guizhou University of Traditional Chinese Medicine, Guiyang, 550001 Guizhou China; 2 Behavioral Neuroscience Laboratory, The First Affiliated Hospital, Guizhou University of Traditional Chinese Medicine, Guiyang, 550001 Guizhou China; 3 College of Acupuncture and Orthopedics, Guizhou University of Traditional Chinese Medicine, Guiyang, 550025 Guizhou China; 4 Department of Neurology, Jinan Geriatric/Rehabilitation Hospital, Jinan, 250013 China; 5 Graduate School of Guangzhou, University of Chinese Medicine, Guangzhou, 510006 China

**Keywords:** Aluminum oxide nanoparticle, Hippocampal CA1 neuron, Memory consolidation, Neuronal activity, Synaptic plasticity

## Abstract

**Background:**

Alumina nanoparticles (aluminaNPs), which are widely used in a range of daily and medical fields, have been shown to penetrate blood-brain barrier, and distribute and accumulate in different brain areas. Although oral treatment of aluminaNPs induces hippocampus-dependent learning and memory impairments, characteristic effects and exact mechanisms have not been fully elucidated. Here, male adult rats received a single bilateral infusion of aluminaNPs (10 or 20 µg/kg of body weight) into the hippocampal region, and their behavioral performance and neural function were assessed.

**Results:**

The results indicated that the intra-hippocampus infusions at both doses of aluminaNPs did not cause spatial learning inability but memory deficit in the water maze task. This impairment was attributed to the effects of aluminaNP on memory consolidation phase through activation of proBDNF/RhoA pathway. Inhibition of the increased proBDNF by hippocampal infusions of p75^NTR^ antagonist could effectively rescue the memory impairment. Incubation of aluminaNPs exaggerated GluN2B-dependent LTD induction with no effects on LTD expression in hippocampal slices. AluminaNP could also depress the amplitude of NMDA-GluN2B EPSCs. Meanwhile, increased reactive oxygen specie production was reduced by blocking proBDNF-p75^NTR^ pathway in the hippocampal homogenates. Furthermore, the neuronal correlate of memory behavior was drastically weakened in the aluminaNP-infused groups. The dysfunction of synaptic and neuronal could be obviously mitigated by blocking proBDNF receptor p75^NTR^, implying the involvement of proBDNF signaling in aluminaNP-impaired memory process.

**Conclusions:**

Taken together, our findings provide the first evidence that the accumulation of aluminaNPs in the hippocampus exaggeratedly activates proBDNF signaling, which leads to neural and memory impairments.

## Introduction

Nanoparticle (NP) is a kind of environmental neurotoxin and the risk of exposure to NPs is enhanced with the increasing use of nanotechnology. Importantly, NPs can penetrate the blood-brain barrier (BBB), blood-gas barrier, and placental barrier [1–3]. Owing to the small size and large surface area, NPs have higher toxicity potential compared with the larger particles of the same material [4, 5]. Therefore, they can accumulate in various organs and tissues and generate toxicological effects [6, 7]. Aluminum oxide nanoparticles (AluminaNPs) have unique physical and chemical characteristics compared to bulk alumina; they are widely used in medicine, water treatment, and manufacturing, raising concerns about the biological safety regarding public health [8, 9]. Although several studies have suggested that aluminaNPs can lead to impair learning and memory ability [10–12], information regarding the neurotoxicity mechanisms of aluminaNPs is currently limited.

The hippocampus, an important research target and model for exploring the cell and molecular mechanisms of learning and memory, exhibits synaptic plasticity changes in these processes [13, 14]. In the mammalian brain, NMDA receptor-dependent synaptic plasticity has been studied in detail in the CA1 region of the hippocampus [15, 16]. Although the most widely studied example of hippocampal long-term potentiation (LTP), which can result in synaptic strengthening, has been widely used as an important indicator of learning and memory variations [17, 18], growing evidence support that GluN2B-dependent long-term depression (LTD) plays central roles in post-learning information sculpting [19–21]. Other findings also indicate that memory consolidation rather than memory acquisition requires the NMDAR-LTD mechanism to modify the hippocampal circuit to store memory [22–24]. Furthermore, it has been well known that abnormal hippocampal LTD in depression, schizophrenia and bipolar disorder leads to deficits in attention, cognition and working memory [25–27]. Therefore, impairments of hippocampal LTD are not only necessary but sufficient to mediate spatial learning and memory. Furthermore, changes in hippocampus neural codes are context-dependent [28]. Hippocampus neurons respond to behavioral performance with dramatic changes in place field reliability, in-field firing rates, and the locations of place fields [29, 30]. Thus, the kind of hippocampus representational organization that has been observed reflects both rate and location remapping [31, 32].

Brain-derived neurotrophic factor (BDNF) is mainly synthesized in the brain and is expressed highly in the cerebral cortex, hippocampus and other parts of the brain [33]. As the precursor form of mature BDNF, proBDNF can act on receptors in the hippocampus and other brain regions related to learning and memory, and regulates synaptic depression by activation of neurotransmitters and its receptor related to these processes [34–37]. Recently, aluminum nanoparticle oral gavage in doses of 5 and 10 mg/kg impaired novel object recognition memory, which coincided with a dose-dependent increase in phosphorylated extracellular signal-regulated kinase (ERK) and cleaved caspase-3 in the hippocampus [38]. Treated with aluminaNPs could also decrease the ratio of active to total level of Akt while increased GSK-3β level in the hippocampus [39]. Furthermore, spatial learning and memory impairment induced by aluminaNPs could be related to mitophagy, as indicated by increases in LC3-II and Beclin-1 activity in the hippocampal CA3 area of mice [11]. Previous studies have also reported that aluminaNPs can cause oxidative stress and inflammatory events in mouse brains [7, 40]. At cellular levels, activation of proBDNF signaling after binding to its receptor p75^NTR^ plays a major role in neuronal apoptosis and the formation of synaptic plasticity through the activation of the downstream RhoA pathway [41–43]. Together, these findings pointed to significant disruptions to neuronal function within the hippocampus, leading to the prediction that they may underlie at least some of the spatial learning deficits and memory disorders observed following the aluminaNP exposure. However, the potential mechanisms have not yet been elucidated.

In this study, we focused on the effects of hippocampal infusions of aluminaNPs on spatial learning and memory and neuronal function important for this processing, especially focusing on the BDNF-NMDA pathway. Moreover, we performed biochemical experiments to test the involvement of proBDNF-p75^NTR^-RhoA signaling pathway and oxidative stress in aluminaNP-induced neurotoxic effects. This provided an important insight into the cognitive function of aluminium-related and other neurodegenerative disorders.

## Materials and methods

### Animal and drug administration

The experiments were performed on male Wistar rats (220–260 g). Animals were obtained from the Beijing Vital River Laboratory Animal Technology Co., Ltd., China. Food and water were made freely available, except when otherwise specified by the experimental design. All experiments were conducted in conformity with the Committee of Guizhou University of Traditional Chinese Medicine for the Care and Use of Laboratory Animals (SCXK-2013-0020).

Aluminium oxide nanoparticles (aluminaNPs) were purchased from Sigma-Aldrich Corporation, with a purity of ≥ 98.5%. The main characteristics of AluminaNPs, including the size, surface area and particle diameter, have been detected in our previous reports [44, 45]. Briefly, it showed that the alumina particle size was distributed from 17 to 76 nm. Its average surface area used in this research was 16.3392 m^2^/g and the average particle diameter was given by 63.8 nm. AluminaNPs were suspended in fresh 0.9% normal saline solution to a concentration of 10.0 or 20.0 µg/µL and bilaterally infused into the hippocampal CA1 region at a dose of 10 or 20 µg/kg of body weight. The doses were quite modest and low compared to previous reports [44–48]. The suspension was sonicated before each infusion. In order to prevent aluminaNP agglomeration, the temperature of the sonicator was kept below 30 °C [46]. For stereotaxic surgery, the animals were anesthetized with isoflurane (induction 5.0%, maintenance 2.5%) and placed in a stereotaxic frame (SN-3, Narishige, Japan) for surgery as described previously [49–51]. Guide cannulae (23-gauge; Plastics One, Inc.) were bilaterally implanted into the CA1 region (AP: −3.5 mm, ML: ± 2.5 mm, DV: −2.0 mm from bregma). Infusions were performed by inserting custom needles (30 Ga, Small Parts Inc.) connected through PE-50 tube into an infusion pump (Harvard Apparatus), extended 1.0 mm from the end of the cannulae. The injections, including aluminaNPs, p75^NTR^ blocker TAT-Pep5 (1.0 ng/µL; Cat#506,181, EMD Millipore) and NMDA-GluN2B antagonist Ro25-6981 (2.0 ng/µL; Cat#1594, Tocris Bioscience), were performed at a rate of 0.5 µL/min for 2 min and the needle was kept in place for 3–5 min to allow diffusion. For all the tests, rats in the control groups received the same volume of vehicle (saline). All the experiments were conducted about 30 min following the aluminaNP infusions. For the co-infusions, the drugs were injected 15 min before aluminaNP infusions. The number of animals used in each group for each test was indicated in the figure legend and more details could be found in Table 1.

**Table 1:** The number of rats in each group for each test

Test	Group							
	Control	AluminaNP-10	AluminaNP-20	AluminaNP 10-TATPep5	TATPep5	Ro25	AluminaNP-10-Int	AluminaNP-10-Exp
Behavioral tests (Fig. 1)								
MWM								
Acquisition phase	16	19	17	–	–	–	–	–
STM test	5	5	5	–	–	–	–	–
LTM test	6	7	6	–	–	–	–	–
Retrieval-LTM	5	7	6	–	–	–	–	–
Open field/ Level press								
–	6	6	6	–	–	–	–	–
Western-blot tests								
mBDNF/CREB /proBDNF/RhoA	5	7	7	–	–	–	–	–
MWM (Fig. 2)								
LTM test	5	5	–	8	5	–	–	–
Slice recordings								
I/O and LTD	5	6	–	6	4	3	6	4
EPSC amplitude /frequency	6	6	–	7	5	–	–	–
Slice ROS								
Superoxide/hydroxyl free radical	5	5	–	5	4	–	–	–
In vivo recordings								
IN/PN neurons	5	7	–	8	5	–	–	–

All experiments were performed on separate groups. Following acquisition training (total *n* = 52), rats from independent subgroups were tested short-term memory (STM, *n* = 15), long-term memory (LTM, *n* = 19) and the retrieval phase of LTM (*n* = 18). The LTM test in the water-maze task was conducted from subgroups without infusions during the acquisition training. The samples used for detecting levels of mBDNF/CREB/proBDNF/RhoA were obtained from the same subgroup. The levels of ROS were detected from the slices without simulations or EPSC recordings

### Behavioral experiments

#### Morris water maze

The Morris water maze (MWM) task has been described previously [52–54]. Briefly, a 150-cm-diameter circular pool was filled with 25 ± 1 °C water opacified with nontoxic black ink. The maze was geographically divided into four equal quadrants and named clockwise I, II, III, and IV. A hidden platform (10-cm-diameter) was located in the center of quadrant III, submerged 2.0 cm beneath the water surface. Fixed, distinctive cues were presented at various locations around the maze. Swimming behavior was monitored by a computerized video tracking system (Ethovision 2.0, Noldus), through which data were collected for offline analysis.

The test was divided into the acquisition phase on day 1 and the probe phase 24 h later. During the acquisition phase, each rat was trained for two consecutive blocks (each block consisted of four trials, 30 s intertrial interval) to find the platform with 5 min intervals. Rats were released into the water individually facing the pool wall from one of four starting points. The order of starting points was used pseudorandom (III, I, IV, II, IV, III, I, II) but the same for all animals. Rats were given 60 s to swim freely to find the platform. A 30 s probe phase, during which the platform was taken out, was conducted 24 h (long-term memory test; LTM) or immediately (short-term memory test; STM) after the training phase. From the tracked swimming traces, a path proximity score was calculated by measuring the distance (cm) between the rat’s position and the platform location [23, 55]. A distance measure was made 10 times per second and averaged across the probe test.

**Fig. 1:**
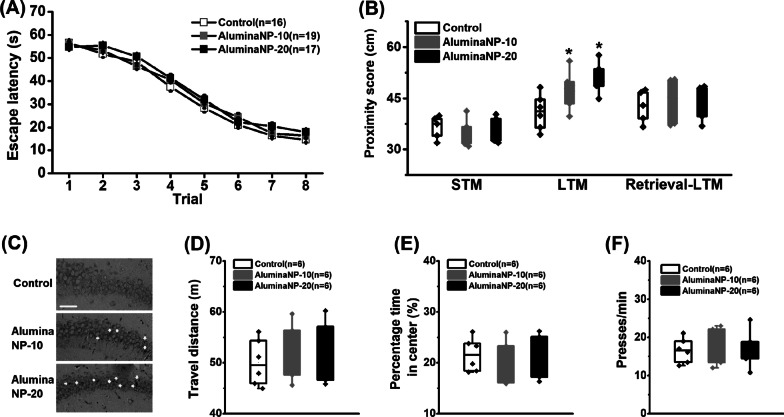
The performance in behavioral tests. **A** Mean escape latency calculated for each trial during the acquisition phase of the MWM task. **B** Effects of aluminaNP on STM, LTM and the memory retrieval during the probe trials. **C** Location of the nanoparticles in the hippocampal CA1 region. The yellow arrows indicated the location of the nanoparticles and the white scale bar presented at the bottom of the photomicrograph indicated 50 μm. **D** Total travel distance and **E** percentage of time spent in the center of the apparatus during the open field test. **F** Press time every min during the lever press test. Data are presented as mean ± SEM. *, *P* < 0.05, versus control group. The number of rats in each group was indicated in each column or legend

**Fig. 2:**
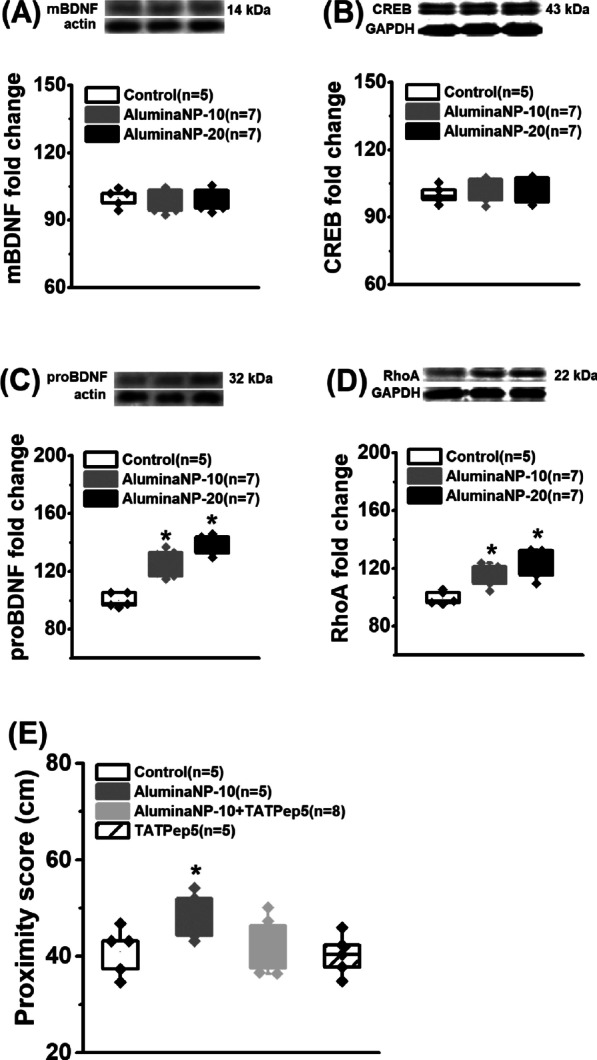
Effects of aluminaNPs on mBDNF-CREB and proBDNF-RhoA signaling pathway. The levels of **A** mBDNF, **B** CREB, **C** proBDNF and **D** RhoA in the hippocampus. *, *P* < 0.05, versus control group. **E** Blocking proBDNF receptor p75^NTR^ activation can mitigate memory deficits. Data are presented as mean ± SEM. *, *P* < 0.05, versus other groups. The number of rats in each group was indicated in each legend

In the memory tests, rats received treatment immediately following the last training trial and then tested STM immediately or LTM 24 h later. In the retrieval memory test, the infusion was performed 30 min before the probe trial. Specifically, we attempted to rule out the possible cumulative effects of aluminaNP infusions. Figure 1: During the acquisition phase, the aluminaNP infusions were conducted on each training day. Figure 2: rats did not receive any infusions during the training phase.

#### Open field test

A separate group of rats (n = 6 for each group) was firstly submitted for the open field test and then the lever press test.

As described previously [56, 57], the open field consisted of a square plastic-made arena (91.5 cm × 91.5 cm × 61 cm). The field was divided into a peripheral region (within 15.25 cm of the walls) and central region (61 cm × 61 cm) of approximately equal area. The test began by placing a single rat in the middle of the arena, and its activity was recorded for 20 min. The parameters analyzed to evaluate locomotor activity in the open field were total distance traveled and time spent in the central areas, which were video-recorded using VersaMax Activity Monitoring System (AccuScan Instruments).

#### Lever press test

Rats were trained to lever press for food pellets in standard operant chambers located inside sound-attenuating boxes (Med Associates). The chambers contained two retractable levers located on either side of a central food trough. As previous studies [58], rats were trained daily 30-min sessions with one of two levers extended randomly when the cue light above the lever was illuminated. The progressive ratio schedule was designed for detecting animals’ motivation. The schedule was progressively changed according to the sequence fixed ratio (FR)-1, FR-15, FR-30, and finally FR-60. Rats were tested in a 30-min session till they reached 10 presses per min on FR-60.

### The observation of nanoparticles

As described previously [23], silver staining was used to detect the location of nanoparticles. Samples were fixed in 4% paraformaldehyde for 30 min and rinsed twice in PBS for 5 min. The slides were immersed in pre-warmed 10% filtered silver nitrate and stained for 30 min. Six milliliters of concentrated ammonium hydroxide were added to the flask containing the silver nitrate solution. Ammoniacal silver was poured onto the slides, which were stained for 15 min, then exchanged for a 1% ammonium hydroxide solution for 3 min and then returned the ammoniacal silver to a flask and added fresh developing solution. The slides were placed in this solution for 5 min and then transferred to 1% ammonium hydroxide solution for 3 min and 5% sodium thiosulfate solution for 5 min. Finally, they were dehydrated and cleared with 95% ethyl alcohol, absolute alcohol, and xylene. The slides were mounted with a resinous medium.

### Western blot assay

Rats were killed by an overdose of urethane and the hippocampus was separated as previously described [59, 60]. Tissue was homogenized in ice-cold lysis buffer (pH 7.4) containing a cocktail of protein phosphatase and proteinase inhibitors (Sigma) and the supernatant was collected. Protein concentrations were detected by bicinchoninic acid assay (Bio-Rad Lab). An equal amount of proteins was resolved by 10–15% SDS-PAGE and then transferred onto PVDF membranes (Pall) for immunoblotting. The membranes were blocked with 5% non-fat skimmed milk for one hour and incubated with the primary mouse anti-proBDNF (1:500; Cat#sc-65,514, Santa Cruz Biotechnology), rabbit anti-mBDNF antibody (1:200; Cat#sc-546, Santa Cruz Biotechnology), rabbit anti-RhoA antibody (1:500, Cat#A13947, Abclonal Biotechnology), rabbit anti-CREB antibody (1:1000, Cat#9197, Cell Signaling Technology), rabbit anti-GAPDH antibody (1:1000, Cat#5174, Cell Signaling Technology) and mouse anti-β-actin antibody (1:20,000; Cat#A5316, Sigma). After three washes with TBST buffer (10 min in each), the membranes were incubated with horseradish-peroxidase (HRP)-conjugated secondary goat anti-mouse (1:2500; Cat#31,430, Thermo Fisher Scientific) or anti-rabbit (1:2500; Cat#31,460, Thermo Fisher Scientific) IgG incubated for one hour. After three washes with TBST buffer, immunoreactivity was detected by ECL Detection Kit (CWBIO).

### In vitro electrophysiological recordings

Hippocampal slices were prepared as previously described [44, 61, 62]. Animals were quickly decapitated and the brains were separated and placed in an ice-cold, oxygenated (95% O_2_ and 5% CO_2_) high-sucrose solution that contained (in mM) sucrose 220, KCl 2.5, MgCl_2_ 6, CaCl_2_ 1, NaH_2_PO_4_ 1.23, NaHCO_3_ 26, and glucose 10, pH 7.4 (with an osmolarity of 300–305 mOsm). Horizontal slices (350 μm in thickness) which included the entire hippocampus were prepared with a vibratome (VT1000S, Leica). After that, slices were moved to a recording chamber mounted on a contrast-enhanced CCD camera (Hamamatsu) equipped with Dodt infrared gradient contrast after a 1 h recovery period. The slices were perfused with a continuous flow of artificial CSF (ACSF, 95% O_2_ and 5% CO_2_) that contained (in mM): NaCl 124, KCl 2.5, MgCl_2_ 2, CaCl_2_ 2, NaH_2_PO_4_ 1.23, NaHCO_3_ 26, and glucose 10, pH 7.4. All experiments were performed at room temperature (22–24 °C).

In LTD recordings, after recovery for at least one hour, the slice was laid in the recording chamber and perfused constantly with ACSF at the rate of 2–3 mL/min as described previously [63–66]. Input/Output (I/O) curves were generated by systematic variation of the stimulus current (0.2–1.0 mA) in order to evaluate synaptic potency. Stimulus pulses were delivered at 0.05 Hz and five responses at each current level were averaged. Low-frequency stimulation (LFS; 900 pulses at 1.0 Hz) induced LTD in CA1 region. To investigate the effects on LTD induction, Ro25-6981 (3.0 µM), slices were incubated with aluminaNP (10.0 µg/µL) or TAT-Pep5 (2.0 µM) 30 min before baseline recording till the LFS accomplishment. To investigate the effects on LTD expression, the infusion of aluminaNP (10.0 µg/µL) was conducted 30 min after LFS accomplishment and continued for 30 min. Initial data measurement was performed in Clampfit 9.0 (Molecular Devices). The slope of field excitatory postsynaptic potentials (fEPSPs) was used to measure synaptic efficacy. The fEPSP slope at every time point was normalized to the mean fEPSP slope during the baseline period. Comparisons among the last 20 min of the 60-min decay were used to analyze.

For the Patch-clamp recording, slices were individually transferred to a recording chamber and the flow rate through the chamber was 2–3 mL/min. Whole-cell voltage-clamp recordings were performed using pipettes with 3–7 M resistance after being filled with pipette solution containing (in mM) K-glu 130, MgCl_2_ 2, HEPES 10, EGTA 3, Na_2_-ATP 2, PH 7.3. All the cells were held at − 70 mV, then slow and fast capacitance compensation was automatically performed. Cells were considered only when the seal resistance was > 500 MΩ and the series resistance (< 30 MΩ) changed < 20% throughout the experiment. Constant negative pressure was applied to form the seal (> 1GΩ) when the recording pipette attached to the membrane. And then suck quickly to rupture the cell membrane and access whole cell configuration. To investigate the contributions of the GluN2B subunit individually, recordings were performed at + 40 mV with 75 µM picrotoxin and CNQX in the bath and the subunit specific current was isolated pharmacologically using 0.5 µM of the GluN2A antagonist PEAQX (NVP-AAM077 tetrasodium hydrate, [[[(1 S)-1-(4-bromophenyl)ethyl]amino](1,2,3,4-tetrahydro-2,3-dioxo-5-quinoxalinyl) methyl] phosphonic acid tetrasodium hydrate). All experiments were carried out at room temperature of ~ 24 °C. All drugs were obtained from Sigma-Aldrich. Only one slice was used for any given experiment.

### Single-unit recording

One week before behavioral training, electrode implantation was conducted using previously reported procedures [56, 67, 68]. Briefly, rats were anesthetized with isoflurane and prepared for surgery. Impedance-measured (200–600 kΩ) microelectrodes were an array of a 4 × 8 matrix using 25-µm-diameter tungsten wires (California Fine Wires, Grover Beach, CA, United States) in a 35-gauge silica tube (World Precision Instruments, Sarasota, FL, United States). An infusion cannula was attached to a silica tube. The proximal open end of the cannula was parallel to electrode tips. Unit activity was amplified (1000–10,000 times) and sampled at 32 kHz and 600–6000 Hz band-pass filters. The firing rates during the probe trial of the MWM test were collected. For all experiments, recording began approximately 15 min following the infusion and the test was conducted 15 min later. Data were acquired on a Digital Cheetah system (Cheetah software; Neuralynx Inc., Bozeman, MT, United States). The rats’ behavior was monitored by a digital ceiling camera (Neuralynx Inc.), and the CCD camera’s signal was fed to a frame grabber (sampling rate, 1 MHz) with the experimental time superimposed for offline analysis.

Spike sorting was performed with offline Neuralynx’s software (SpikeSort 3D), using a combination of KlustaKwik, followed by manual adjustment of the clusters (Klusters software package). Briefly, multiple parameters were used to determine the clusters with the most often used combination of spike height, trough, and energy, associated with the waveforms. As in the previous studies [23, 34, 58], units were graded for quality and classified as pyramidal neurons and fast-spiking (FS) interneurons. The firing rate was collected during the whole probe trial of the MWM test and the mean frequency was obtained when animals approached the hidden platform within 40 cm.

### Determination of oxidative stress in the hippocampal slices

Hippocampal slices were prepared and incubated in the recording chamber as described above. After about 30 min incubation, the hippocampal slices were rinsed in 0.1 M phosphate buffer (pH 7.4) and homogenized with ice-cold saline to be 10% (w/v) homogenates. The mixtures were homogenized using a glass homogenizer for 5 min on ice and centrifuged at 3000 rpm at 4 °C for 15 min. The supernatant was collected and stored at − 70 °C for the biochemical tests.

The levels of superoxide anion radical and hydroxyl free radical were detected according to the methods described in the references using commercial ELISA kits (Institution of Nanjing Jiancheng Biological Engineering, Nanjing, China). Firstly, add prepared samples and standards, antibodies labeled with enzymes, reacting 60 min at 37 °C. Secondly, plate washed for 5 min, adding chromogen solution, reacting 10 min at 37 °C. Finally, add the stop solution and measure the OD value for 10 min. The protein levels of samples were measured by the Coomassie Brilliant Blue G-250 method with bovine serum albumin as standard.

### Data acquisition and statistical analysis

The experimental data were represented as mean ± S.E.M. Escape latencies of the MWM, I/O curve and time coursing changes of fEPSP slope and neuronal activity during the behavioral test were analyzed with Repeated Measures ANOVA. Others were analyzed with one-way ANOVA. Post hoc analyses were performed with Tukey’s test where appropriate. The probability value of less than 0.05 was considered to be statistical significance.

## Results

### Effects of alumina nanoparticles on behavioral performance

The results obtained from the acquisition and probe phases of the MWM test are presented in Fig. 1A, B, respectively. A two-way repeated ANOVA test revealed a significant trial effect on the escape latency (Fig. 1A; *F*_(7, 343)_ = 4.57, *P* < 0.001) but no treatment effect (*F*_(2, 49)_ = 0.12, *P* = 0.887), indicating all groups could gradually learn to locate the hidden platform. In the probe tests, alumina nanoparticles were infused into the CA1 regions immediately following the acquisition training in the STM test (STM), 30 min following the acquisition training in the consolidation test (LTM), or 30 min prior to the LTM test in the retrieval test (LTM-retrieval) (Fig. 1B). No treatment effect was found in the STM (*F*_(2, 12)_ = 0.21, *P* = 0.814) or memory retrieval test (*F*_(2, 15)_ = 0.15, *P* = 0.862) but a significant treatment effect on the memory consolidation test (*F*_(2, 16)_ = 3.97, *P* = 0.040). Subsequent post-hoc comparisons revealed the proximity score of both aluminaNPs-10 and aluminaNPs-20 was higher than control group (control vs. aluminaNPs-10 or aluminaNPs-20, both *P* < 0.05), indicating impairments of spatial memory consolidation in aluminaNP-treated rats. Thirty min following the infusions, the accumulation of nanoparticles in the hippocampal tissue was also observed in both aluminaNP-treated groups (Fig. 1C), which could confirm the cognitive deficits and neuronal dysfunction (see below) were caused by the nanoparticles.

A one-way ANOVA test revealed no main effects on the total exploration distance (Fig. 1D; *F*_(2, 15)_ = 0.16, *P* = 0.853) or percentage time stay in the center area (Fig. 1E; *F*_(2, 15)_ = 0.11, *P* = 0.896) during the open field test, or the press time during the lever press test (Fig. 1F; *F*_(2, 15)_ = 0.13, *P* = 0.879). Therefore, the impairments in probe trials are not due to the effects on locomotion or motivation defects.

### Activation of proBDNF-RhoA pathway involves in spatial memory deficits

The results showed that aluminaNP infusions did not change the expression of mBDNF (Fig. 2A; *F*_(2, 16)_ = 0.52, *P* = 0.604) or CREB (Fig. 2B; *F*_(2, 16)_ = 0.59, *P* = 0.565) expression. However, the levels of proBDNF (Fig. 2C; *F*_(2, 16)_ = 4.77, *P* = 0.024; control vs. aluminaNPs-10 or aluminaNPs-20, both *P* < 0.05) and RhoA (Fig. 2D; *F*_(2, 16)_ = 5.36, *P* = 0.017; control vs. aluminaNPs-10 or aluminaNPs-20, both *P* < 0.05) were abnormally elevated in the hippocampus. Blocking the activation of p75^NTR^ by its inhibiter TAT-Pep5 could effectively decline the elevation of proximity score induced by the infusions of aluminaNP at the dose of 10.0 µg/kg of body weight (Fig. 2E; *F*_(3, 19)_ = 3.49, *P* = 0.036; aluminaNPs-10 + Pep5 vs. aluminaNPs-10, *P* < 0.05), without alterations in control group (control vs. Pep5, *P* > 0.05). Together, these results demonstrate that aluminaNP-mediated proBDNF-p75^NTR^-RhoA signaling is associated with the memory impairment.

### Alumina nanoparticles inhibit LTD induction and GluN2B-EPSC amplitude, and induce oxidative stress

According to the behavioral findings, in parallel experiments we measure whether aluminaNP-induced deficits of memory consolidation correlated with disruption of hippocampal synaptic function. To minimize the number of animals used, we only tested aluminaNP effects at the low dose of 10.0 µg/µL.

**Fig. 3:**
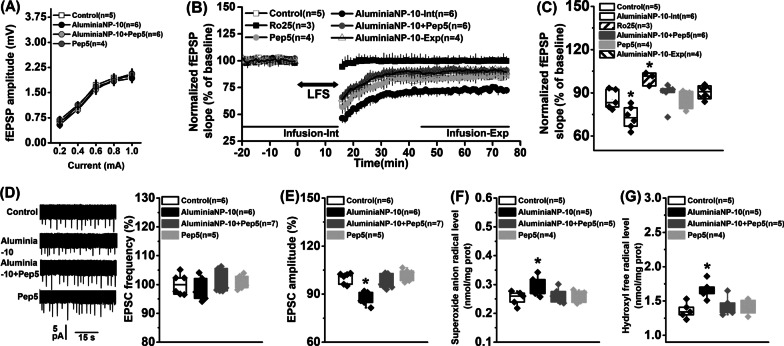
Effects of aluminaNP on neuronal function in the hippocampal slices. **A** Input–output synaptic function. **B** Characteristic time courses of fEPSP slope. The bidirectional arrow indicated the application of LFS. **C** Time coursing changes in fEPSPs slope. The magnitude of LTD was determined as responses between 41 and 60 min after LFS. **D** A typical consecutive sample trace of NMDA-GluN2B EPSC from each group (left) and the frequency of NMDA-GluN2B EPSCs (right). **E** The amplitude of NMDA-GluN2B EPSCs. The levels of **F** superoxide anion radical and **G** hydroxyl free radical in the homogenates of hippocampal slices. Data are presented as mean ± SEM. *, *P* < 0.05, versus other groups. The number of rats in each group was indicated in each legend

A repeated measures ANOVA on the I/O curve revealed a statistical current effect (Fig. 3A; *F*_(4, 76)_ = 4.19, *P* = 0.004) but no treatment effect (*F*_(3, 19)_ = 0.18, *P* = 0.909). In the LTD recording, stimulation of Schaffer collaterals evoked a basal fEPSP in hippocampal CA1, and LFS-induced LTD of the stimulated synapses for at least 1 h (Fig. 3B; treatment effect: *F*_(5, 22)_ = 6.36, *P* < 0.001). It was found that LTD was significantly enhanced in the aluminaNP-treated group compared to that in the control (Fig. 3C; *F*_(5, 22)_ = 4.29, *P* = 0.007; aluminaNP-10-Int vs. control, *P* < 0.05). Inactivation of p75^NTR^ signaling drastically suppressed the increased fEPSP slope (aluminaNP-10-Int vs. aluminaNP-10 + Pep5, *P* < 0.05), with no alteration in control slices (Pep5 vs. control, *P* > 0.05). However, the incubation of aluminaNP during the expression of the LTD lacked effects on the fEPSP slope (aluminaNP-10-Exp vs. control, *P* > 0.05). The selective antagonist Ro25-6981 effectively blocked the LFS-induced LTD, verifying that the GluN2B-dependent LTD was induced in this study. Furthermore, there was a significant treatment effect on amplitude (Fig. 3E; *F*_(3, 20)_ = 4.46, *P* = 0.015) but not frequency (Fig. 3D; *F*_(3, 20)_ = 0.41, *P* = 0.748) of the EPSCs. The aluminaNP-10 group showed significantly low EPSC amplitude compared with control group (aluminaNP-10 vs. control, *P* < 0.05). However, the infusion of Pep5 mitigated the reduction in amplitude (aluminaNP-10 vs. aluminaNP-10 + Pep5, *P* < 0.05) with no changes in control slices (control vs. Pep5, *P* < 0.05). Additionally, we have also tested HFS((8 pulses at 100 Hz for 6 s repeated 30 times)-induced long-term potentiation (LTP), but no significant difference in the fEPSP slope was found among aluminaNP-10, aluminaNP-20 and control groups (*F*_(2, 14)_ = 0.17, *P* = 0.845; aluminaNP-10(*n* = 7): 127.31 ± 1.96; aluminaNP-20(*n* = 6): 123.58 ± 1.62; control(*n* = 4): 126.49 ± 1.22). These findings confirm that aluminaNP-induced increase in proBDNF affects the induction of LTD by enhancing postsynaptic GluN2B activation.

The effects of aluminaNPs on the levels of superoxide anion radical and hydroxyl free radical were determined and the results were presented in Fig. 3F, G, respectively. After incubation with aluminaNPs for 30 min, both levels of superoxide anion radical (*F*_(3, 15)_ = 3.77, *P* = 0.033; control vs. aluminaNPs-10, *P* < 0.05) and hydroxyl free radical (*F*_(3, 15)_ = 4.04, *P* = 0.027; control vs. aluminaNPs-10, *P* < 0.05) were enhanced significantly. Furthermore, inactivation of p75^NTR^ signaling could effectively reduce superoxide anion radical (aluminaNPs-10 + Pep5 vs. aluminaNPs-10, *P* < 0.05) and hydroxyl free radical (aluminaNPs-10 + Pep5 vs. aluminaNPs-10, *P* < 0.05) levels. No statistical difference was found between control and Pep5 groups (Pep5 vs. control, both *P* > 0.05).

### Alumina nanoparticles weak neuronal correlate of long-term memory in the hippocampus

**Fig. 4:**
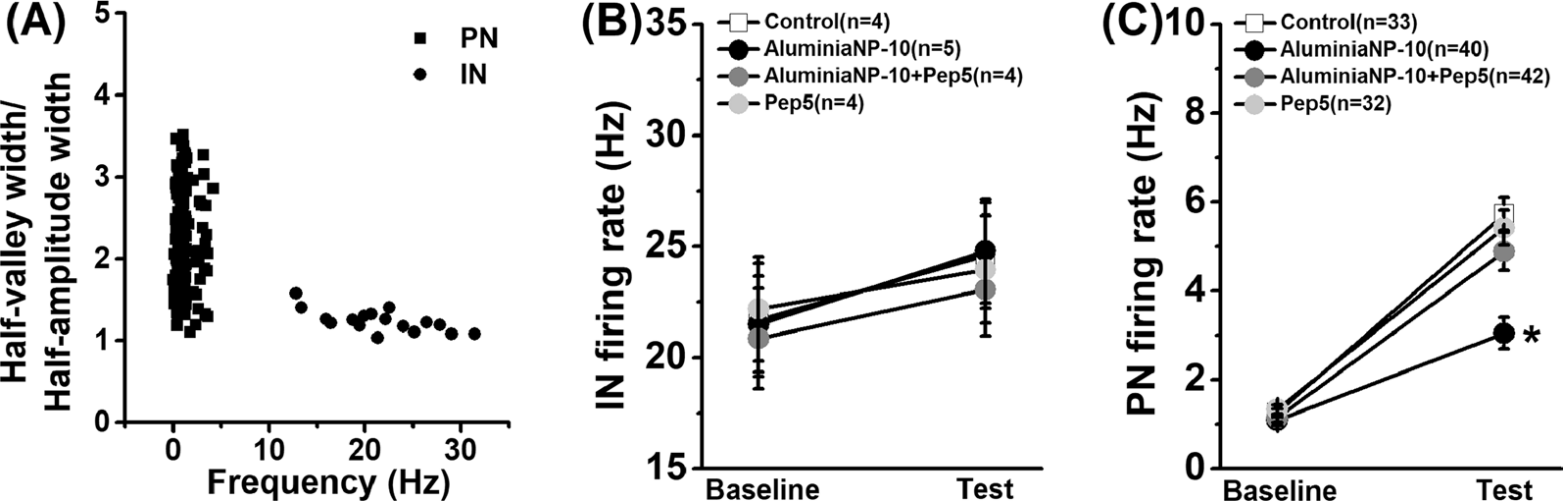
AluminaNP weakens neuronal correlate of memory behavior. **A** Distribution of mean firing rate and half-valley to half-peak ratio of each unit from the recording. **B** Firing rate of fast-spiking interneurons (IN) during the probe test. **C** Firing rate of pyramidal neurons (PN) during the probe test. Data are presented as mean ± SEM. *, *P* < 0.05, versus other groups. The number of rats in each group was indicated in each legend

Neuron spike trains were classified by waveform shape (half-valley width/half-amplitude width) and spiking patterns (firing frequency) (Fig. 4A). One hundred forty-seven units were isolated from the hippocampus of twenty-five rats during memory probe test. Wide-wave form neurons were classified as pyramidal neurons (PN; 33 from five rats of control group, 89.2% of group population; 40 from seven rats of aluminaNP group, 90.9% of the population; 42 from eight rats of aluminaNP + Pep5 group, 89.4% of the population; 32 from five rats of Pep5 group, 88.9% of the population) while narrow-wave form neurons were classified as fast-spiking interneurons (IN; 4 from five rats of control group, 10.8% of the population; 4 from seven rats of aluminaNP group, 9.1% of the population; 5 from eight rats of aluminaNP + Pep5 group, 10.6% of the population; 4 from five rats of Pep5 group, 11.1% of the population). For the fast-spiking interneurons, a repeated measures ANOVA revealed no main treatment effect (Fig. 4B; *F*_(3, 13)_ = 0.08, *P* = 0.969). Compared with control group, aluminaNP group showed a significant decline in firing rate of pyramidal neurons during the probe test (Fig. 4C; *F*_(3, 143)_ = 3.95, *P* = 0.001; aluminaNP-10 vs. control, *P* < 0.05) with no difference in basal frequency (aluminaNP-10 vs. control, *P* > 0.05). Infusions of Pep5 during the memory consolidation stage could obviously facilitate neuronal correlate in the aluminaNP group (aluminaNP-10 vs. aluminaNP-10 + Pep5, *P* < 0.05) but not affect the firing rate of controls (control vs. Pep5, *P* > 0.05). Our findings support that the increase in hippocampal proBDNF levels induced by aluminaNPs disrupts memory consolidation, leading to weakening the neuronal correlate of long-term spatial memory.

## Discussion

Learning and memory play a critical role in cognitive processes. In the brain, aluminum predominantly accumulates in the hippocampus and frontal cortex, regions are known to involve in the etiology of Alzheimer’s disease (AD) [69, 70]. Sub-acute aluminaNP supplementation causes neurodegeneration and apoptotic neuronal loss [40, 71, 72], along with cognitive dysfunction, including attention, learning ability and memory formation [11, 39]. Moreover, previous studies have found its potent immunotoxicity [73] and genotoxicity [74]. In this study, our findings indicate the presence of a correlation between aluminaNP accumulation in the hippocampal region and the potential dangers to spatial learning and memory. It was supported by the findings that intra-hippocampal infusions of aluminaNPs (10 µg/kg or 20 µg/kg body weight) decreased the time needed to accurately locate the platform with the training, suggesting that rats in both groups learned to find the platform. Nevertheless, the aluminaNP-treated groups had a lower spatial performance than the control group did, which was revealed by the probe test results. This deficit was further confirmed by the effects of aluminaNP on memory consolidation, but not memory retrieval. These observations are in agreement with the results from intranasal treatment with 0.1 mg/kg aluminaNP, which displayed low neural toxicity, as indicated by no alternation in spacial learning behaviors and expression of beta-amyloid precursor protein (Aβ) [75]. Nevertheless, in the olfactory bulb, they found massive ED1 expressions, a marker of microglia/macrophage activation. The BDNF-mediated increase in ED1 staining was previously shown to correlate with inflammatory activation [76], suggesting that macrophage activation may be directly related to the age-associated increase in BDNF signaling. Consistently, we found the abnormal up-regulation of the proBDNF/RhoA signaling pathway without change in the mBDNF or CREB level in the hippocampus. Previous studies showed that proBDNF had an effect on learning strategy [29] and extinction of contextual fear memory but not on learning ability [49]. It is most possible that aluminaNP interfered with PHF8 protein expression and function, inhibit H3K9me2 demethylation, and lead to elevate proBDNF expression [77]. More recently, hippocampal glutamate metabolism disorder was found to be induced by treatment with aluminaNPs via activating IFN-γ/ASK1/JNK signaling pathway [72]. More importantly, the RhoA-JNK pathway can be activated after proBDNF binds to the p75^NTR^ receptor, which in turn induces oxidative damage and promotes apoptosis, thus affecting synaptic plasticity [41, 78]. Therefore, our evidence implies that the activation of proBDNF-RhoA signaling by aluminaNP infusions is an important factor for spatial memory deficits.

ProBDNF is transported anterogradely, sorted to dense-core synaptic vesicles in the CA1 region and released upon depolarization, thus, reducing neuronal excitability and synaptic strength [42, 79, 80]. For example, blocking hippocampal proBDNF-p75^NTR^ signaling by infusion of TAT-Pep5 at a dose of 4.0 ng/µL disrupted place-strategy behavior accompanying the suppression of GluN2B-mediated LTD induction but not its expression at the Schaffer collateral-CA1 pathway [23, 29]. Prior studies indicated that LTD-null mice lacking serum response factor failed to habituate to novel objects in an object-recognition task [81]. Interestingly, these mice also displayed poor spatial memory in the MWM. Indeed, artificial intervention facilitation of LTD induction is often accompanied by a decline in learning and memory capacity [82, 83], supporting the hypothesis that LTP and LTD are not independent but an entity in modulating efficiency of spatial cognition [84–86]. Several studies have documented that the acute neurotoxic effect of aluminaNPs was attributed to their ability to cause mitochondrial dysfunction and disruption of brain energy homeostasis, such as Na^+^, K^+^-ATPase activity [87], which play key roles in metabolism in neurons and postsynaptic calcium response [88]. These results imply that aluminaNP-induced ATPase dysfunction has profound effects on the modulation of neuronal plasticity and NMDA-evoked currents [89, 90]. Specifically, aluminaNP-induced reactive oxygen species can modify the biological properties of the membrane [71, 91, 92], impair normal cellular function [93, 94], and generate new oxidized products that can damage other macromolecules [93, 95, 96], thus leading to neuronal hyperactivity and synaptic dysfunction. LTP inhibits GSK-3β and the activation of GSK-3β is required for induction of LTD, indicating a role for GSK-3β in metaplasticity [97]. Interestingly, the memory impairing effect of aluminaNPs was attributed to the deregulation of Akt/GSK-3β signaling in the hippocampus [39]. Furthermore, aluminaNPs and aluminum ions can also disturb the neurotransmission metabolism of aspartate, tyrosine, glutamate and serine [98, 99].

Population dynamics in hippocampal place cells are extremely important for the biological control of spatial memory [100]. The involvement of the proBDNF-p75^NTR^ axis in controlling pyramidal neuron excitability and maintaining network homeostasis in the adult central nervous system (CNS) has been recently reported [101]. In this study, the increased activity of proBDNF in the aluminaNP-infused rats inhibited the firing frequency of the hippocampal pyramidal neurons but not fast-spiking interneurons during the memory probe test. Consistent with these findings, aluminaNPs alter rhythmic and synchronized activities in the antennal lobe of Drosophila [102]. Studies in rats have shown that the formation of memory is related to prolonged phosphorylation and activation of hippocampal CREB, which by binding to a critical calcium response element within the BDNF gene activates BDNF transcription to modulate synaptic transmission [103, 104]. Similarly, proBDNF in vivo tends to exert a negative effect on neuronal activity-dependent processes [105] and inhibit the mnemonic property of entorhinal pyramidal neurons [106]. This finding seems to overlap with the report that direct recordings of fast-spiking interneurons in dentate gyrus cells revealed that mBDNF reduced neural excitability and depressed their action potential firing, whereas proBDNF had no effect [107]. The best-understood explanation is that proBDNF receptor p75^NTR^ is not expressed in interneurons [108], in accordance with the inability of proBDNF to modulate GABAergic activity [107]. Therefore, the impairment of memory consolidation induced by aluminaNP is due to the enhanced proBDNF expression in the hippocampal regions leading to exaggerated synaptic depression and weakened neuronal correlate of LTM performance.

Changes in both neurotrophins and their receptor levels occur during aging and neurodegeneration. In aged rats, proBDNF levels are elevated in the hippocampus while precursor nerve growth factor (proNGF) is elevated both in the hippocampus and cortex [109]. Increased proBDNF levels were also found in human and mouse late-life depression, Parkinson’s disease (PD) and Down’s syndrome brains, disorders characterized by learning and memory deficits and neuronal degeneration similar to AD [110–112]. The activation of proBDNF-p75^NTR^ signaling in the aged hippocampus could not only inhibit neural proliferation but also induce apoptosis in differentiated neurons [105, 113]. Our study revealed a similar elevation of proBDNF and ROS levels in the acute aluminaNP-exposed rat’s hippocampus. What we have to note is that, in this study, the rats were still in the medium age during all the tests, so the increase in proBDNF level should be associated with the effect of aluminaNP rather than aging.

Although administration routes (injection, inhalation, subcutaneous, etc.) in mammals have been shown to affect their toxic effects [7, 114], aluminaNPs are more likely to accumulate in the brain compared with other organs [115]. Moreover, multiple sub-brain regions involved in maintaining stress, anxiety, learning, and memory ability can be affected by exposure to aluminaNPs [12, 71, 116]. For example, aluminaNPs administered orally (100 mg/kg) to rats were shown to obviously affect motor neurons (motor neurons of medulla oblongata and midbrain red nucleus) and neurons with inhibitory action on certain motor neurons (Purkinje cells of cerebellum) [117]. Following exposure to aluminaNPs at the dose of 32 mg/kg, oxidative stress was observed in both the prefrontal and hippocampus of rats, which presented alteration in long-term memory but not short-term memory [116]. The accumulation of aluminaNPs in adult zebrafish brain tissue could result in symptoms similar to AD, including progressive learning and memory disorders and anxiety-like symptoms [12]. Additionally, aluminaNPs are also detectable in striatum and olfactory bulb [71, 75], which play distinct roles in decision making and the formation of an olfactory memory respectively [34, 118, 119]. To exclude the effects from other brain regions and detect the specific effects on the hippocampus to show the potential associated mechanisms, the intra-hippocampal infusion was selected for this study. As we observed, administration of aluminaNPs to rats orally as a single dose at 500 and 1500 mg/kg or cutaneously at 1000 and 2000 mg/kg significantly changed oxidative markers in brain [120]. M’rad et al. administered aluminaNP intravenously to rats with a lower dose of 20 mg/kg body for 4 days and found that aluminum content 0.89 ± 0.14 µg/g in hippocampus [99]. AluminaNP treatments markedly altered the brain electrolyte contents, e.g., Na^+^ and K^+^ in the brains of rats and mice 24 h after administration through intravenous (30 mg/kg), intracarotid (2.5 mg/kg) or intracerebroventricular (20 µg) routes [46]. Intragastric gavage of the rats with 32 mg/kg aluminaNPs for 2 months increased the aluminum content to 675.7 ± 37.7 µg/g in hippocampus tissue [116], which is consistent with the doses selected in the current study. Furthermore, our doses are comparable with others in mammalian and human studies [121–123] and environmental risks [124, 125].

BDNF actions are mediated by ligand-specific Trk receptors and p75^NTR^, with a third receptor, sortilin, binding the immature or pro-neurotrophins [126, 127]. Specifically, the interaction of proBDNF with sortilin and p75^NTR^ on the cell surface is required to initiate cell death [128]. Although we have confirmed the involvement of proBDNF-p75^NTR^-RhoA signaling in the neurotoxic effects of aluminaNPs, further research can be done to prove the possibility of this hypothesis. Furthermore, the neuronal redox state, such as the increased oxidative tone displayed by neurons during aging, is significantly influenced by neuronal BDNF [129, 130]. Hence, identifying the cellular and molecular entities engaged in activity-dependent cross-talk between neuronal proBDNF and ROS signaling and unraveling their effects on LTD need to be further investigated.

## Conclusions

In conclusion, our findings extend the understanding of the effects of intra-hippocampal aluminaNP on memory deficits, which are mostly attributed to its disruptive effect on memory consolidation. The increased proBDNF expression leads to exaggerating hippocampal LTD and reducing the amplitude of EPSC while the blockage in its receptor p75^NTR^ can effectively mitigate cognitive impairments and synaptic function. Furthermore, the deteriorated memory process could be attributed to the interference by proBDNF-mediated neuronal excitability during the spatial LTM test. In general, our study investigates a new pathway of neurotoxic aluminaNP to disrupt the memory process and evaluates their potential mechanism. Further investigation into the influence of NPs on the central nerve pathway and neurodegeneration is necessary. Therefore, we suggest that the inclusion of NPs in food and medical products should be studied further to provide a better understanding of the potential negative impacts of them on CNS.

## Data Availability

The data that support the findings of this study are available from the corresponding author upon reasonable request.
